# Clinical, CT, and MR Findings for the Pretreatment Diagnosis of Primary Epithelial Tumors of the Lacrimal Gland

**DOI:** 10.2174/0115734056474643260813050851

**Published:** 2026-08-24

**Authors:** Yu-Yun Chang, Kuei-Hong Kuo, Hsin-Wei Wu, Chia-Hung Wu, Te-Ming Lin, Jung-Hsuan Chen, Chung-Han Yang, Feng-Chi Chang

**Affiliations:** 1 Division of Medical Imaging, Far Eastern Memorial Hospital, Banciao, Taipei, Taiwan; 2 Department of Radiology, Taipei Veterans General Hospital, Taipei, Taiwan; 3 School of Medicine, National Yang Ming Chiao Tung University, Taipei, Taiwan; 4 Department of Radiology, Taichung Veterans General Hospital, Taichung, Taiwan; 5 Neuroscience Research Center, Taipei Medical University Hospital, Taipei, Taiwan

**Keywords:** Lacrimal gland, Adenoma, Adenoid cystic carcinoma, Magnetic resonance imaging (MRI), Computed tomography (CT), Primary epithelial tumors

## Abstract

**Introduction/Background:**

The diagnosis of benign and malignant primary epithelial tumors of the lacrimal gland (PET-LG) is challenging. This study was designed to obtain a pretreatment diagnosis of adenoma and Adenoid Cystic Carcinoma (ACC) of the LG based on clinical and imaging features.

**Materials and Methods:**

A retrospective analysis was conducted on 18 patients with histologically confirmed PET-LG (6 adenomas and 12 ACCs). Demographic data, clinical outcomes, and imaging findings were collected. MR findings included lesion size, signals, contrast enhancement patterns, DWI/ADC values, and evidence of orbital involvement. For ACC cases, CT findings were compared with MRI findings using pathological evidence as the reference. Statistical analyses were performed to assess differences between the adenoma and ACC groups.

**Results:**

ACCs showed a more aggressive imaging pattern than adenomas. Compared with adenomas, ACCs were larger at presentation and more frequently demonstrated vivid contrast enhancement, orbital wall destruction, and extraocular muscle involvement. Among these findings, orbital wall invasion and extraocular muscle involvement were the most robust imaging features associated with ACC. In contrast, adenoma more commonly showed smooth orbital wall erosion or remodeling. For assessing bone invasion in ACC, MRI showed higher sensitivity and accuracy than CT, whereas CT remained useful for evaluating cortical bone changes.

**Discussion:**

Imaging findings can be used to diagnose benign and malignant PET-LG. MRI plays a pivotal role in identifying these imaging features for clinical staging, but CT remains valuable for detecting bone erosion.

**Conclusion:**

We suggest applying both CT and MR of the orbit as imaging techniques to enhance pretreatment planning.

## 1 INTRODUCTION

Primary Epithelial Tumor of Lacrimal Gland (PET-LG) accounts for approximately 22% of Lacrimal Gland (LG) tumors. Among them, Adenoid Cystic Carcinoma (ACC) is the most common malignant epithelial neoplasm, whereas adenoma is the most common benign epithelial tumor [1]. At initial presentation, their symptoms are usually nonspecific, *e.g*., pain, a protruding mass, ptosis, diplopia, *etc*. ACC and adenoma of the LG also share some similar neuroimaging features that make their differentiation challenging [2]. CT is usually the initial and most common imaging tool because of its accessibility and good ability to evaluate bony invasion. However, CT has limitations in the diagnosis of subtle soft tissue lesions or early bone marrow lesions. Although MRI provides clear soft tissue assessment, only a limited number of studies have presented diagnostic clues for the MR characteristics of ACC and adenoma [3]. Both tumors exhibit similar signal changes and enhancement on MR images after contrast administration, although ACC is slightly more obviously enhanced than adenoma.[4]. Even the DWI and ADC value calculations were reported to have equivocal results [5]. Only bony destruction and intracranial invasion are strongly associated with the diagnosis of advanced ACC [6].

Although definite surgical excision is recommended for PET-LG, ACC and adenomas have different behaviors and outcomes. Compared with ACC in the salivary gland, ACC in the LG is highly recurrent and has worse outcomes [7]. The ACC of LG is associated with a 10% survival rate and with 20% rates of recurrence, growth, and distant metastasis. In comparison with simple surgical resection of adenoma, extensive exenteration combined with chemotherapy and radiotherapy is believed to improve the outcome of ACC. Hence, accurate pretreatment diagnosis is of paramount importance for the early surgical planning of PET-LG. This retrospective study was designed to develop methods for pretreatment diagnoses of adenoma and ACC of the LG by their clinical and MR findings and thus to improve patient outcomes.

## 2 METHODS

### 2.1 Patients

From 2004 to 2024, a total of 154 patients with suspected lacrimal gland lesions were identified from our institutional imaging archive. Patients were eligible for inclusion if they had histopathologically confirmed PET-LG and available preoperative CT and MRI examinations. Patients were excluded if they lacked presurgical MRI, had no pathological confirmation, or had non-PET-LG lesions, such as lymphoma, IgG4-related disease, or idiopathic orbital inflammatory disease. The final study cohort consisted of 18 patients, including 12 patients with ACC and 6 patients with adenoma (Fig. **1**). Clinical characteristics, surgical records, adjuvant treatment, tumor recurrence or progression, survival, and imaging findings were retrospectively reviewed.

### 2.2 CT

All the patients underwent presurgical contrast-enhanced orbital CT one month before MR examination. Orbital CT was performed on 128-slice MDCT scanners using thin-slice acquisition (0.5–0.125 mm) with 3 mm soft tissue reconstructions. Axial images were acquired parallel to the orbitomeatal line; coronal images were perpendicular. Multiplanar reformates (axial, coronal, and sagittal) were generated. Scanning included both precontrast and postcontrast phases, with contrast-enhanced images obtained 70–80 seconds after injection.

### 2.3 MRI

Orbital MRI was performed with a 1.5T clinical MR scanner (GE Medical Systems) with an 8-channel head coil. The protocol included axial and coronal pre-contrast T1-weighted images (T1WI); axial, sagittal, and coronal pre-contrast T2-weighted images (T2WI); axial and coronal post-contrast T1WI; and axial diffusion-weighted imaging (DWI). The two-point Dixon technique was used to generate fat-suppressed images for the pre-contrast and post-contrast axial T1WI. DWI was performed in the axial plane using a single-shot spin-echo echo-planar imaging sequence with b-values of 0 and 1000 s/mm^2^. Apparent diffusion coefficient maps were automatically generated by the MRI scanner. Post-contrast sequences were obtained immediately after intravenous administration of a gadolinium-based contrast agent at a dose of 0.1 mmol/kg.

### 2.4 Image Analysis

All MR images were reviewed for tumor location, size, signal intensity, enhancement pattern, diffusion characteristics, and perilesional changes. Tumor signal intensity was compared with that of the adjacent normal extraocular muscle [8]. Contrast enhancement on T1-weighted imaging was considered vivid when the degree of enhancement was greater than that of the contralateral normal lacrimal gland or uninvolved lacrimal gland (Figs. **2**-**4A-**F****).

### 2.5 Tumor Location and Size Measurement

Tumor location was classified according to involvement of the superficial lobe, deep lobe, or both lobes of the lacrimal gland. The superficial lobe was defined as the smaller and more palpable portion of the lacrimal gland, whereas the deep lobe was defined as the portion extending deep to the orbital septum. Tumor size was measured as the maximum tumor diameter on T2-weighted images in any imaging plane.

### 2.6 Diffusion-weighted Imaging and ADC Measurement

Diffusion restriction was assessed visually on DWI and ADC maps. Absolute ADC values (ADCmax/min) were measured by manually placing Regions of Interest (ROIs) of approximately 10 mm^2^ on the ADC maps using the hospital Picture Archiving and Communication System (PACS) workstation. The tumor ROIs were placed in the solid tumor component showing the lowest ADC signals, while avoiding necrotic, cystic, hemorrhagic, or calcified areas. ADCmax and ADCmin were recorded (Figs. **2F**, **3F**, **4F**).

### 2.7 ADC Ratio Calculation

To reduce the influence of inter-scan signal variability, an additional ROI was placed in the normal-appearing ipsilateral temporal white matter on the same or corresponding axial section (Figs. **2F**, **3F**, **4F**). The ADC ratio was calculated as the ratio of tumor ADC to ipsilateral temporal white matter ADC.

### 2.8 Perilesional Imaging Features

We evaluated perilesional changes, including bony erosion (Fig. **3G**, bony invasion (Figs. **2B-D**, **4C, D**), adjacent muscle involvement (Fig. **2D**, **4D**) and proptosis (Fig. **2B**). Bony erosion was defined as smooth thinning, scalloping, or remodeling of the inner periorbital cortical bone with a relatively well-defined margin. Bony invasion was defined as direct tumor invasion into the periorbital bone on T2WI and postcontrast T1WI (Figs. **2B-D**, **4B-D**). It was further correlated with surgical findings and pathology reports when available [1]. Extraocular muscle involvement was defined as tumor infiltration into the adjacent extraocular muscle rather than simple mass effect or indentation (Figs. **2D**, **3D**, **4D**). Proptosis was assessed by measuring the distance from the interzygomatic line to the anterior surface of the globe on cross-sectional MR images parallel to the optic nerve and lens (Fig. **2B**). Severe proptosis was defined as a difference in distance >2 mm [9]. Intratumoral calcification was recorded by comprehensively evaluating T1WI and T2WI, which presented low signals on both T1WI and T2WI, and was further confirmed by presurgical orbital CT findings.

All CT and MRI interpretations were reviewed by two readers: CFC (Neuroradiologist with more than 30 years of experience) and CYY(Radiologist with 4 years of experience). Imaging features were assessed according to predefined operational criteria (Table **S1**). Discrepancies between the two readers were resolved by consensus.

### 2.9 Statistics

All the statistical analyses were performed with RStudio (version 2026.01.2+418). PET-LGs are rare. All eligible patients identified from 2004 to 2024 were included, yielding 18 cases. As this retrospective study had a fixed sample, no *a priori* power calculation was performed. Analyses were exploratory, with exact methods and 95% confidence intervals used where appropriate; nonsignificant results should not be interpreted as equivalence. Continuous variables were summarized as mean ± standard deviation with 95% confidence intervals. Given the small sample size, between-group comparisons of continuous variables were performed using the Mann-Whitney U test. Categorical variables are summarized as counts and percentages. *P*-values were analyzed using Fisher’s exact test. Holm-Bonferroni correction was applied to adjust for multiple comparisons. Adjusted *p*-values of less than 0.05 were considered to indicate statistical significance. Diagnostic performance metrics, including sensitivity, specificity, positive predictive value, negative predictive value, and accuracy, were reported with 95% confidence intervals calculated using exact binomial methods. Among ACCs, ROC analysis was performed to evaluate the ability of tumor size to predict orbital wall bone destruction. The area under the ROC curve was calculated, and the size cutoff was selected from the ROC coordinates as the threshold providing 100% specificity with the highest available sensitivity. Because of the limited sample size and lack of external validation, the ROC-derived cutoff was considered exploratory and hypothesis-generating.

### 2.10 Ethics

This retrospective study was reviewed and approved by the Institutional Review Board of Taipei Veterans General Hospital, Taipei, Taiwan (IRB No. IRB2022-07-020BC). The requirement for written informed consent was waived due to the retrospective nature of the study and the use of anonymized patient data. All procedures involving human participants were conducted in accordance with the ethical standards of the institutional research committee and the Declaration of Helsinki.

## 3 RESULTS

### 3.1 Demographic Features

The characteristics of the 6 patients with adenoma and 12 patients with ACC are summarized in Table **1**. Women were predominant in both the adenoma and ACC groups, but the difference was not significant [83.3% (5/6) *vs*. 91.7% (11/12), *p*=1.000].

### 3.2 Clinical Outcomes

In the ACC group, disease progression was observed in 44.4% of evaluable patients (4/9), with a mean interval between surgery and recurrence or progression of 27.7±15.0 months (95% CI 3.7-51.7). Compared with the adenoma group, patients with ACC more frequently received adjuvant chemotherapy [0% *vs*. 80% (8/10)] and radiotherapy [16.7% (1/6) *vs*. 83.3% (10/12)]. All the patients with adenomas survived, while 2 patients with ACC died.

### 3.3 MRI Features

The MRI features of the 6 adenomas and the 12 ACCs are provided in Table **1**. On pretreatment MRI, ACCs tended to be larger than adenomas, although the difference did not remain statistically significant [24.4 ± 9.0 *vs*. 37.4 ± 13.5, *p* = 0.407]. ACCs also more frequently showed vivid enhancement on contrast-enhanced T1WI than adenomas [50% (3/6) *vs*. 100% (12/12), *p* = 0.343]. The raw data for calculating the ADC were accessible for the 4 adenomas and 11 ACCs. There was no significant difference in the ADC value or ADC ratio between adenomas and ACCs. With respect to peritumoral involvement, extraocular muscle involvement was significantly more frequently observed in ACCs than in adenomas [0% (0/6) *vs*. 83.3% (10/12), *p*=0.026]. Orbital wall bony destruction was also significantly more frequent in ACCs than in adenomas [0 *vs*. 83.3% (10/12), *p* = 0.026]. Orbital wall bone erosion was more frequently observed in adenomas than in ACCs [83.3% (5/6) *vs*. 8.3% (1/12), *p* = 0.059]. In ROC curve analysis, tumor size showed moderate discriminatory ability for predicting orbital wall bone destruction, with an AUC of 0.788. A cutoff of >43 mm yielded 50% sensitivity and 100% specificity. However, this cutoff was derived from a small retrospective cohort and should be interpreted as exploratory rather than as a validated clinical threshold.

## 4 DISCUSSION

Primary epithelial tumors of the lacrimal gland are uncommon, and pretreatment differentiation between adenoma and adenoid cystic carcinoma remains clinically important because of their distinct surgical strategies and prognosis [10, 11] (Table **2**). In this small retrospective cohort, adenoid cystic carcinomas showed a more aggressive imaging pattern than adenomas, particularly with respect to orbital wall destruction and extraocular muscle involvement. After adjustment for multiple comparisons, these two findings remained significant, suggesting that direct invasion of adjacent structures may be among the most useful MRI features for differentiating malignant from benign lacrimal gland epithelial tumors.

Bone invasion is a key imaging feature in lacrimal gland ACC because it directly affects staging and treatment planning. In our ACC cohort, MRI showed higher accuracy than CT for detecting bone invasion (90% *vs* 70%, Table **S2**), suggesting that contrast-enhanced MRI may better delineate marrow invasion that is less apparent on CT. This distinction is clinically relevant because periosteal and bone invasion upgrade the T category in the AJCC 8th edition staging system and may influence the extent of surgical management. CT remains valuable for evaluating cortical remodeling or erosion; therefore, combined CT and MRI assessment provides complementary information for pretreatment evaluation [10] (Table **S3**).

The pattern of orbital wall change may also help guide the radiologic impression. Adenomas more commonly produced smooth remodeling or erosion of the adjacent orbital wall, whereas ACCs more frequently showed destructive bone invasion. However, because the difference in bone erosion did not remain statistically significant after correction for multiple comparisons, this finding should be interpreted as supportive rather than definitive. In contrast, orbital wall destruction remained significantly associated with ACC and may therefore represent a more robust marker of malignant behavior.

Extraocular muscle involvement was another imaging feature that remained significantly more frequent in ACC after multiple-comparison correction. This finding reflects the infiltrative behavior of ACC and may help distinguish malignant invasion from the mass effect typically seen in adenomas. Because MRI provides superior soft-tissue contrast, it is particularly useful for assessing tumor extension into adjacent muscles, orbital soft tissues, and possible dural or perineural spread.

Other MRI findings, including larger tumor size and stronger contrast enhancement, were more commonly observed in ACCs but did not remain statistically significant. These findings may still contribute to the overall imaging impression, especially when combined with invasive features, but they should not be considered independent diagnostic markers in this small cohort.

Previous work by Zhang *et al*. [12] reported that ADC values and ADC ratios may help differentiate pleomorphic adenoma from malignant lacrimal gland tumors. In contrast, our study did not identify significant ADC differences between adenoma and ACC. This discrepancy may reflect the limited number of cases with available DWI, differences in tumor composition, and variability in acquisition parameters, b-values, partial volume effects, and ROI placement. Therefore, ADC-related findings in the present study should be interpreted as exploratory and require validation in larger cohorts with standardized DWI protocols.

## 5 STUDY LIMITATIONS

This study has several limitations. First, the cohort was small because of the rarity of PET-LG; therefore, negative findings may reflect limited statistical power rather than true absence of difference. Second, although Holm correction was applied to reduce the risk of type I error from multiple comparisons, the corrected results should still be interpreted cautiously. Third, the ROC-derived tumor size cutoff for predicting bone destruction was exploratory and should not be used as a definitive clinical threshold without external validation. Fourth, the imaging was reviewed by two readers with different levels of experience, and the readers were not blinded to the final pathological diagnosis, which may have introduced interpretation bias. Formal inter-observer agreement analysis was not performed. Finally, the retrospective design and heterogeneous imaging acquisition may have influenced image interpretation and quantitative ADC measurements.

## CONCLUSION

In conclusion, orbital wall destruction and extraocular muscle involvement are the most robust imaging features associated with ACC. MRI is particularly useful for evaluating bone marrow invasion, extraocular muscle invasion, and possible dural or perineural spread, whereas CT remains valuable for assessing cortical bone erosion and remodeling. Other findings, including larger tumor size, stronger enhancement, and bone erosion patterns, may provide supportive diagnostic information but should be interpreted cautiously. A combined CT and MRI approach remains valuable for pretreatment assessment, with CT contributing to evaluation of cortical bone changes and MRI improving assessment of invasive soft-tissue and marrow involvement.

## Figures and Tables

**Fig. (1):**
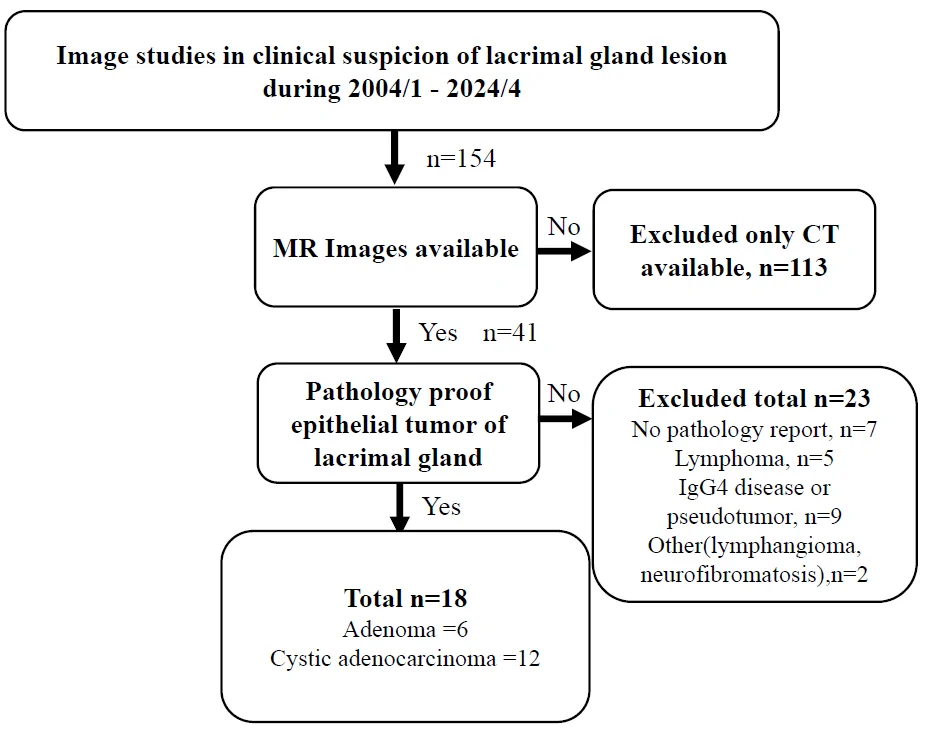
Patient selection flowchart.

**Fig. (2):**
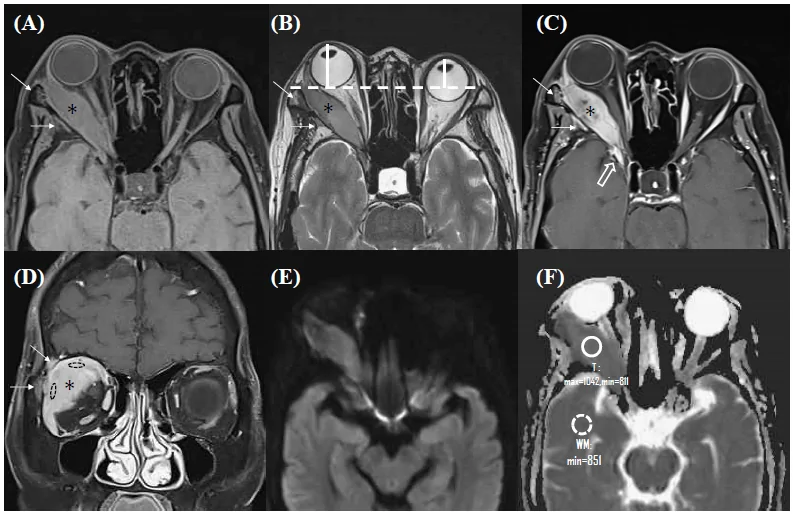
A 71-year-old woman presented with proptosis and was diagnosed with ACC. Orbital MRI with fat-saturated axial T1WI (**A**) and axial T2WI (**B**), contrast-enhanced T1WI in the axial/coronal view (**C**, **D**), DWI (**E**), and ADC (**F**). ACC presents as isointense on T1WI (asterisk in A) and heterogeneous hyperintensity on T2WI (asterisk in B). Assessment of proptosis by measuring the distance from the interzygomatic line (dashed line in B) to the anterior surface of the globe on a cross-sectional image parallel to the optic nerve and lens. The difference in the distance measurement was 8.2 mm (>2 mm), defined as severe proptosis. (B) Increased contrast enhancement of the tumor compared with that of the contralateral normal LG (not shown) (C, D) and the enhanced lesion with intracranial invasion (hollow arrow in C). Tumor invasion into the lateral orbital wall was noted on T2WI, Postcontrast T1WI (arrow in B, C and D), and extraocular muscle involvement (star sign in D). Diffusion restriction was shown by DWI (b=1000) (E). The tumor ROI was positioned on the homologous area with the lowest level within the lesion on the ADC (F). The additional ROI was placed on the normal-appearing homologous area of the ipsilateral temporal white matter in the same section. The ADCmax was 1042 mm^2^/s, the ADCmin was 811 mm^2^/s, and the calculated ADC ratio was 1.22 (F).

**Fig. (3):**
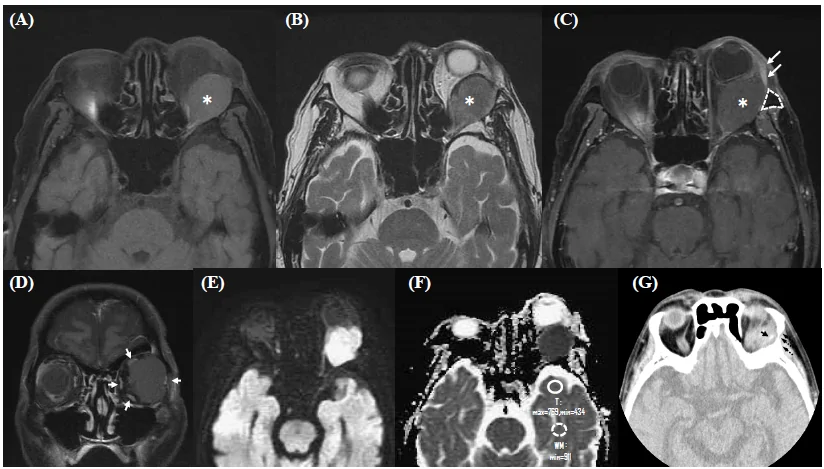
An 84-year-old woman presented with proptosis and was diagnosed with oncocytic adenoma. Orbital MR sequences of T1WI (**A**), T2WI (**B**), contrast-enhanced T1WI in axial/coronal view (**C**, **D**), DWI (b=1000) (**E**), ADC (**F**), and orbital CT (**G**). The tumor presents isointensity on T1WI and T2WI (asterisk in A, B). The tumor presents non-vivid enhancement in contrast-enhanced T1WI (asterisk in C), and the enhanced intraocular muscle is indented outward by the tumor (arrow in C, D). The orbital wall is free of tumor invasion (dotted line in C). Equivocal diffusion restriction on DWI (E). The measured ADCmax is 769 mm^2^/s, whereas the measured ADCmin is 434 mm^2^/s. The calculated ADC ratio was 0.84 (F). Intratumor calcification (black arrow in G) was confirmed by a preoperative orbital CT scan, which clearly demonstrated bone erosion by the tumor (dotted arrow in G).

**Fig. (4):**
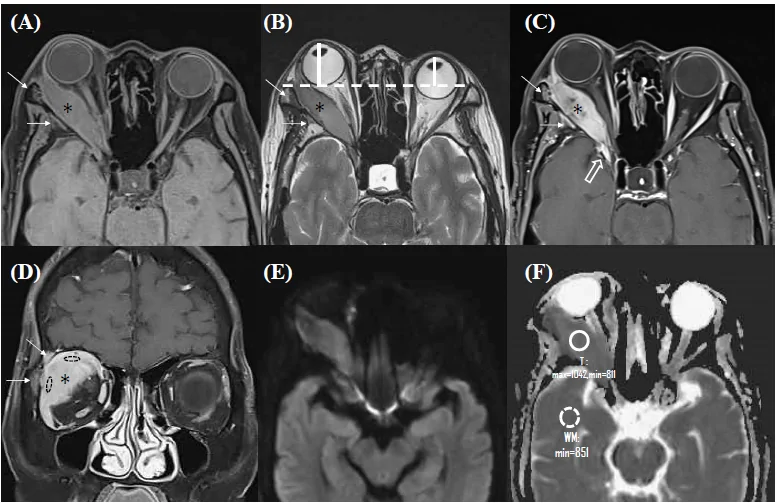
A 42-year-old woman diagnosed with ACC underwent orbital MRI comprising T1WI (**A**), T2WI (**B**), contrast-enhanced T1WI in axial and coronal views (**C**, **D**), DWI (b=1000) (**E**) and ADC mapping (**F**). The tumor is isointense on T1WI (A) and heterogeneous on T2WI (B). Intratumor calcification (asterisk in A, B, C) at the central site, which is confirmed by orbital CT (not shown), is characterized by low signal on both T1WI and T2WI along with nonenhancement on contrast-enhanced T1WI (A, B, C). Bony invasion along the anterior clinoid process (arrow in C) causes intracranial invasion. Bony invasion also involved the superior lateral orbital wall (dotted line in C). Intracranial invasion along the frontal dura is demonstrated by coronal contrast-enhanced T1WI (arrow in D), with evident infiltration into the orbital wall. Equivocal diffusion restriction is observed on DWI (E). The measured ADCmax is 910 mm^2^/s, whereas the measured ADCmin is 690 mm^2^/s. The calculated ADC ratio is 1.09 (F).

**Table 1:** Demographic features of the 18 patients with epithelial tumor of the lacrimal gland.

-	**Adenoma (N=6)**	**ACC (N=12)**	**Adjusted-*p*-value**
**Sex (M/F) (%)**	1(16.7)/5(83.3)	1(8.3)/11(91.7)	1
**Age (YR)**	48.6±21.2 (95% CI 26.3-70.9)	57.4±15.2 (95% CI 41.7-67.1)	1
**Surgical Results**						
**Total – No. (%)**	4(66.7)	10(83.3)	1
**Partial – No. (%)**	2(33.3)	2(16.7)	
**Adjuvant Treatment**						
**Chemotherapy – No. (%)**	0	8 (66.7)	0.192
**Radiotherapy – No. (%)**	1(16.7)^+^	10(83.3)	0.516
**Outcome**						
**Recurrence/Metastasis – No. (%)** ^A^	0	5(45.5)	1
**Follow Up Period (MON)**	28.1±26.3 (95% CI 0.5-55.8)	51.6±49.3 (95% CI 9.7-75.1)	1
**Mortality – No (%)**	0	2(16.7)	1
**MR Features**						
**Size (MM)**	24.4±9.0 (95% CI 14.9-33.9)	37.4±13.5 (95% CI 29-45.9)	0.407
**Side (Right/Left) - No. (%)**	2(33.3)/4(66.6)	6(50)/6(50)	1
**Location (Superficial/Deep/Both) - No. (%)**	1(16.6)/2(33.3)/3(50)	0/6(50)/6(50)	1
**Signal Change**						
**T2 Hyperintensity – No. (%)**	4(66.7)	8(66.7)	1
**T2 Iso- or Hypointensity – No. (%)**	2(33.3)	4(33.3)	
**T1 Hyperintensity – No. (%)**	2(33.3)	0	0.578
**T1 Iso- OR Hypointensity – No. (%)**	4(66.7)	12(100)	
**Well Enhancement T1/C –No. (%)**	3(50)	12(100)	0.343
**No or Poor Enhancement T1/C–No. (%)**	3(50)	0	
**ADC MAX** ^B^ **– 10^-6^MM^2^/S**	1250.7±322.9 (95% CI 736.9-1764.5)	1265.4±382.9 (95% CI 1008.2-1522.7)	1
**ADC MIN** ^B^ **– 10^-6^MM^2^/S**	906.7±355.9 (95% CI 340.4-1473.0)	797.0±247.5 (95% CI 630.7-963.3)	1
**ADC Ratio** ^B^ **– Tumor/Ipsilateral Temporal White Matter**	1.36±0.35 (95% CI 0.8-1.93)	1.39±0.36 (95% CI 1.15-1.63)	1
**Calcification Content**	1(16.6%)	2(16.6%)	1
**Peri-Lesional Change**						
**Muscle Involvement**	0	10(83.3%)	**0.026**
**Orbital Wall Involvement**						
**Bone Erosion**	5(83.3%)	1(8.3%)	0.059
**Bone Destruction**	0	10(83.3%)	**0.026**
**Intracranial Invasion**	0	4(33.3%)	1
**Proptosis**						
**No Proptosis**	3(50)	2(16.7)	1
**Proptosis (0-2MM)**	1(16.7)	3(25)	
**Severe Proptosis(>2MM)**	2(33.3%)	7(58.3%)	

^*^Other symptoms, including diplopia as an initial complaint, in one pleomorphic adenoma case.

^+^Pathology revealed oncocytic adenoma; according to the literature concerning the salivary gland, oncocytoma may transform malignantly. Hence, 50 Gy of adjuvant radiotherapy was applied in 25 fractions after partial excision [13].

^A^A patient with ACC was newly diagnosed and underwent exenteration with adjuvant radiotherapy and chemotherapy for a follow-up period < 3 months. Two cases of ACC were lost after diagnosis; hence, we excluded them from the calculation.

^B^DWI and ADC were not performed in the two pleomorphic adenoma patients or one ACC patient; thus, they were excluded from the calculation.

**Table 2:** Literature review of MRI characteristics of lacrimal gland adenoma and adenoid cystic carcinoma.

Study/Refs.	Year		Patient Number	Iso- or Hypo-intensity T1WI	Hyperintensity T2WI	Well Enhancement T1C	ADC (x10^-3^ mm^2^/sec)	ADC Ratio	Muscle Involvement		Bone Erosion	Bone Destruction	Intracranial Invasion
Adenoma													
Gündüz *et al*. [14]	2003	3		Iso: 3 (100)^+^	Heterogeneous: 3 (100)^+^	3 (100)	-	-		-	2 (67)	0	-
Reza Vagefi *et al*.[15]	2007	3		-	-	-	-	-		-	1 (33.3)	-	-
Zhang *et al*.[12]	2014	18		-	-	-	1.37±0.22	1.85±0.34		-	-	-	-
Adenoid cystic carcinoma													
Gündüz *et al*.[14]	2003	3		Iso: 3 (100)^+^	Homogeneous: 3 (100)	3 (100)	-	-		-	-	3 (100)	-
Williams *et al*.[6]	2010	14		-	-	-	-	-		-	-	11(78.6)	-
Qin *et al*.[16]	2011	17		13 (100%)*	6 (46.2%)*	Homogeneous: 5 (38.4) Heterogeneous: 8 (61.6)	-	-		-	-	14 (82.7)	-
Wang et el.[17]	2012	6		-	-	-	-	-		-	-	2 (33.3)	-
Zhang *et al*.[12]	2014	2		-	-	-	1.0±0.06	1.27±0.17		-	-	-	-

**Note:** *Signal compared with the gray matter.

^+^Signal compared with extraocular muscle and gray matter.

## Data Availability

All the data and supportive information is provided within the article.

## LIST OF ABBREVIATIONS

ACC: Adenoid cystic carcinoma
ADC: Apparent diffusion coefficient
AJCC: American Joint Committee on Cancer
AUC: Area under the receiver operating characteristic curve
CI: Confidence interval
CT: Computed tomography
DWI: Diffusion-weighted imaging
IRB: Institutional Review Board
LG: Lacrimal gland
MDCT: Multidetector computed tomography
MR: Magnetic resonance
MRI: Magnetic resonance imaging
PACS: Picture archiving and communication system
PET-LG: Primary epithelial tumor of the lacrimal gland
ROC: Receiver operating characteristic
ROI: Region of interest
T1WI: T1-weighted imaging
T2WI: T2-weighted imaging

## STANDARDS OF REPORTING

STROBE guidelines were followed.
