# Supplementary material for: Clinical, CT, and MR Findings for the Pretreatment Diagnosis of Primary Epithelial Tumors of the Lacrimal Gland
Source: Curr Med Imaging. 2026 Aug 24;22:E15734056474643. doi: 10.2174/0115734056474643260813050851 (PMC13736225; doi:10.2174/0115734056474643260813050851)
Supplement: Supplementary file 1 [file CMIM-22-E15734056474643_SD1.pdf]

BENTHAM SCIENCE

CrossMark

Current Medical Imaging

Content list available at: https://benthamscience.com/journals/cmir

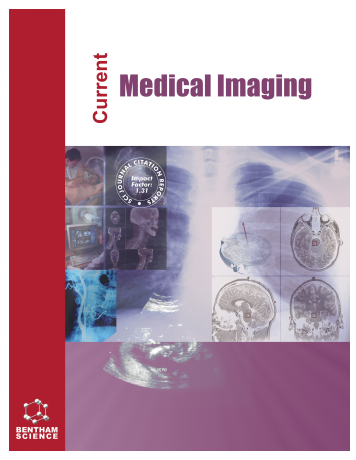

Supplementary Material

Clinical, CT, and MR Findings for the Pretreatment Diagnosis of Primary Epithelial Tumors of the Lacrimal Gland

Yu-Yun Chang[1], Kuei-Hong Kuo[1,3], Hsin-Wei Wu[2,3], Chia-Hung Wu[2,3], Te-Ming Lin[2,3], Jung-Hsuan Chen[2,3,4,#], Chung-Han Yang[2,3,*] and Feng-Chi Chang[2,3,5,*]

[1]*Division of Medical Imaging, Far Eastern Memorial Hospital, Banciao, Taipei, Taiwan*
[2]*Department of Radiology, Taipei Veterans General Hospital, Taipei, Taiwan*
[3]*School of Medicine, National Yang Ming Chiao Tung University, Taipei, Taiwan*
[4]*Department of Radiology, Taichung Veterans General Hospital, Taichung, Taiwan*
[5]*Neuroscience Research Center, Taipei Medical University Hospital, Taipei, Taiwan*

Table S1. Operational definitions of CT and MRI imaging features evaluated in primary epithelial tumors of the lacrimal gland.

Imaging feature	Imaging modality	Operational definition/assessment criteria
Tumor location	MRI	Tumor location was classified according to involvement of the superficial lobe, deep lobe, or both lobes of the lacrimal gland.
Tumor size	MRI, mainly T2WI	Tumor size was measured as the maximum tumor diameter on T2-weighted images in any imaging plane.
Signal intensity on T1WI	MRI, T1WI	Tumor signal intensity was visually compared with that of the adjacent normal extraocular muscle and categorized as hyperintense, isointense, or hypointense.
Signal intensity on T2WI	MRI, T2WI	Tumor signal intensity was visually compared with that of the adjacent normal extraocular muscle and categorized as hyperintense, isointense, or hypointense.
Vivid enhancement	MRI, post-contrast T1WI	Vivid enhancement was defined as tumor enhancement greater than that of the contralateral normal lacrimal gland or uninvolved lacrimal gland.
Diffusion restriction	MRI, DWI and ADC map	Diffusion restriction was assessed visually on DWI and ADC maps. Restricted diffusion was considered present when the lesion showed high signal on DWI with corresponding low signal on the ADC map.
ADCmax / ADCmin	MRI, ADC map	ADC values were measured by manually placing approximately 10 mm² ROIs in the solid tumor component showing the lowest ADC signal, while avoiding necrotic, cystic, hemorrhagic, or calcified areas. ADCmax and ADCmin were recorded.
ADC ratio	MRI, ADC map	ADC ratio was calculated as the ratio of tumor ADC to ipsilateral temporal white matter ADC measured on the same or corresponding axial section.
Bone erosion/remodeling	CT and MRI	Bone erosion was defined as smooth thinning, scalloping, or remodeling of the inner periorbital cortical bone with a relatively well-defined margin, without definite marrow replacement or destructive invasion.
Bone invasion/destruction	MRI, T2WI and post-contrast T1WI; correlated with CT	Bone invasion was defined as direct tumor extension into the periorbital bone, with abnormal marrow signal or enhancing soft-tissue infiltration within the orbital wall. Findings were correlated with surgical findings and pathology reports when available.
Extraocular muscle involvement	MRI, mainly post-contrast T1WI and T2WI	Extraocular muscle involvement was defined as direct tumor infiltration into the adjacent extraocular muscle, rather than simple displacement, compression, or indentation caused by mass effect.

DOI: 10.2174/0115734056474643260813050851, 2026, *22*, e15734056474643

Intracranial invasion	MRI, post-contrast T1WI	Intracranial invasion was defined as direct tumor extension beyond the orbital wall into the intracranial compartment, including dural involvement or intracranial soft-tissue extension.
Dural invasion	MRI, post-contrast T1WI	Dural or perineural invasion was considered present when there was abnormal enhancement or tumor extension along the dura, orbital apex, superior orbital fissure, cavernous sinus, or expected course of adjacent cranial nerves.
Proptosis	MRI axial image parallel to optic nerve and lens	Proptosis was assessed by measuring the distance from the interzygomatic line to the anterior surface of the globe. Severe proptosis was defined as a side-to-side difference greater than 2 mm.
Intratumoral calcification	CT and MRI	Calcification was suspected on MRI when intratumoral foci showed low signal on both T1WI and T2WI and was confirmed by presurgical orbital CT.

Table S2. Diagnostic performance of CT and MRI in detecting bone invasion in lacrimal gland adenoid cystic carcinoma.

**Image features**		**CT**	**MR**
Image evidence of bone invasion	Sensitivity	71.4%	100%
	Specificity	66.7%	66.7%
	PPV	83.3%	87.5%
	NPV	50.0%	100%
	Accuracy	70.0%	90.0%

Note: Sensitivity = True Positive/(True Positive + False Negative). Specificity = True Negative/(False Positive + True Negative). PPV = True Positive/(True Positive + False Positive). NPV = True Negative/(True Negative + False Negative). Accuracy = (True Positive + True Negative)/All samples.

Table S3. Bone involvement in lacrimal adenoid cystic carcinoma: Pathological findings compared with CT/MR imaging features and the impact on clinical staging.

				**Imaging**							
**Case**	**Age (yr)**	**Sex**	**Size (mm)**	**CT feature of bone invasion and clinical staging by CT**		**MR feature of bone invasion and clinical staging by MR**		**Pathology finding of bone invasion**	**Pathology staging**	**Outcome**	**Follow-up (months)**
1	40	F	44	+	T3b	+	T4c	+	T4cN0M0	R, M, A	23
4	64	F	42	-	T3a	-	T3a	-	T3a	A	89
5	64	F	50	+	T3c	+	T3c	+	T3c	A	22
8	36	F	45	+	T3c	+	T3c	NA	NA	L	1
9	81	F	16.5	-	T1a	+	T1c	+	T2c	E	3
10	32	F	27	-	T2a	-	T2a	-	T2a	R, A	132
11	57	F	29	-	T2a	+	T2c	+	T2c	R, A	118
12	58	M	38	+	T2b	+	T2c	+	T2c	A	1
13	75	F	24.4	-	T2a	+	T2c	NA	NA	L	-
16	54	F	58	+	T3b	+	T3c	+	T3c	M, A	4
17	57	F	32	+	T2b	+	T2c	+	T2c	E, M	52
18	71	F	52	+	T3b	+	T3c	-	T3	A	22

Note: A: Alive, R: Recurrence, L: Loss, M: Distant metastasis, E: Expired, NA: Not available.

© 2026 The Author(s). Published by Bentham Science Publisher.

This is an open access article distributed under the terms of the Creative Commons Attribution 4.0 International Public License (CC-BY 4.0), a copy of which is available at: https://creativecommons.org/licenses/by/4.0/legalcode. This license permits unrestricted use, distribution, and reproduction in any medium, provided the original author and source are credited.

HOW TO CITE:
Chang Y, Kuo K, Wu H, Wu C, Lin T, Chen J, Yang C, Chang F. Clinical, CT, and MR Findings for the Pretreatment Diagnosis of Primary Epithelial Tumors of the Lacrimal Gland. Curr Med Imaging, 2026; 22: e15734056474643.
http://dx.doi.org/10.2174/0115734056474643260813050851
